# Assessing Methanogenic Archaeal Community in Full Scale Anaerobic Sludge Digester Systems in Dubai, United Arab Emirates

**DOI:** 10.2174/1874285801812010123

**Published:** 2018-04-30

**Authors:** Munawwar A. Khan, Poojabahen G. Patel, Arpitha G. Ganesh, Naushad Rais, Sultan M. Faheem, Shams T. Khan

**Affiliations:** 1 Department of Life and Environmental Sciences, College of Natural and Health Sciences, Zayed University, P.O.Box: 19282, Dubai, UAE; 2 School of Life Sciences, Manipal University, Dubai International Academic City, P.O.Box 345050, Dubai, UAE; 3 Department of Agricultural Microbiology, Aligarh Muslim University, Aligarh, 2002002, UP. India

**Keywords:** Anaerobic digestion, Archaea, Fluorescence *in situ* hybridization, Methanogens, Quantitative Polymerase Chain Reaction, Sequencing methods, Hydrogenotrophic

## Abstract

**Introduction:**

Anaerobic digestion for methane production comprises of an exceptionally diverse microbial consortium, a profound understanding about which is still constrained. In this study, the methanogenic archaeal communities in three full-scale anaerobic digesters of a Municipal Wastewater Treatment Plant were analyzed by Fluorescence *in situ* hybridization and quantitative real-time Polymerase Chain Reaction (qPCR) technique.

**Methods & Materials:**

Fluorescence *in situ* hybridization (FISH) was performed to detect and quantify the methanogenic *Archaea* in the sludge samples whereas qPCR was carried out to support the FISH analysis. Multiple probes targeting domain archaea, different orders and families of Archaea were used for the studies.

**Results and Discussion:**

In general, the aceticlastic organisms *(Methanosarcinaceae & Methanosaetaceae)* were more abundant than the hydrogenotrophic organisms *(Methanobacteriales, Methanomicrobiales, Methanobacteriaceae & Methanococcales)*. Both FISH and qPCR indicated that family *Methanosaetaceae* was the most abundant suggesting that aceticlastic methanogenesis is probably the dominant methane production pathway in these digesters.

**Conclusion:**

Future work involving high-throughput sequencing methods and correlating archaeal communities with the main operational parameters of anaerobic digesters will help to obtain a better understanding of the dynamics of the methanogenic archaeal community in wastewater treatment plants in United Arab Emirates (UAE) which in turn would lead to improved performance of anaerobic sludge digesters.

## 1 INTRODUCTION

Anaerobic digestion plays an immensely significant role in the treatment of highly concentrated organic wastes. This process is beneficial in two simple yet important aspects: Firstly, the process is anaerobic thereby eliminating the need for aeration and rendering it as a cost-effective strategy. Secondly, in addition to diminishing the pollution load, anaerobic digestion converts complex organics into methane (biogas) [1]. It is accomplished *via *the interaction between a diverse consortium of microorganisms, in four distinct stages: hydrolysis, acidogenesis, acetogenesis and methanogenesis [2]. The harmonized activity of the microbial consortia *i.e.*, hydrolyzing/acidifying bacteria (acidogens) and methane generating Archaea (methanogens) is crucial from the perspective of process efficiency [3]. Anaerobic digestion has been comprehensively explained in terms of process engineering but our knowledge about the microbial communities is still scarce which could be a major reason resulting in process failure in full scale anaerobic digestion facilities [4].

The final stage of Anaerobic digestion *i.e.*, methanogenesis is performed by obligate anaerobic Archaea and account for over 90% of CH_4_ generated on Earth [5]. Methanogenesis is accomplished *via *three different pathways namely a) hydrogenotrophic (H_2_/CO_2_ used for CH_4_ synthesis) b) acetoclastic (involves transfer of methyl group from acetate to tetrahydrosarcinapterin and finally to coenzyme M (CoM)) c) methylotrophic methanogenesis (production of methyl CoM by utilizing methyl groups from methanol and methylamines (mono-, di-, and trimethylamine). These three pathways converge at a common final step in which methyl CoM is converted into methane by an enzymatic complex ubiquitous in all methanogens *i.e.*, methyl coenzyme M reductase [6]. These methanogens categorized into five main orders within the Archaeal domain *i.e.*, Methanobacteriales, Methanopyrales, Methanomicrobiales, Methanosarcinales and Methanococcales are able to use only a minimal number of thermodynamically suitable substrates for methane production [7, 8]. Methanogenic archaea have proven to be extremely challenging in terms of cultivation in the laboratory due to their requirement of very peculiar environmental conditions [9, 10].

There have been quite a few culture-independent techniques employed for the investigation of the methanogenic consortia like: a) Fluorescence *In situ* Hybridization (FISH) [11-13] b) Terminal Restriction Fragment Length Polymorphism (T-RFLP) [14] c) community fingerprinting by Denaturing Gradient Gel Electrophoresis (DGGE) [15] d) real-time quantitative PCR (qPCR) [16-18] e) Single Strand Conformation Polymorphism (SSCP) [19]. As suggested in the past studies conducted in several countries, anaerobic sludge digester (ASD) treatment systems in the UAE are susceptible to several operational problems, potentially reducing their overall efficiency. Excessive competition and growth of problematic microbial community members are widely recognized as the main cause of reduced efficiency of ASD systems. On the other hand, the fluctuations of operational parameters of ASD systems might also affect stability and balance of microbial communities. Therefore, more detailed and fundamental understanding of microbial populations is required for effective long-term control of ASD conditions. Molecular techniques have shown promise for gaining a better understanding of microbial community members in ASD treatment systems and quantitative data provided by molecular techniques such, as FISH [11-13] and Q-PCR [16-18] have been successfully utilized in several studies to validate engineered models and to optimize biogas production and for this reason these two molecular approaches have been used in the current study.

There have been many studies dealing with the characterization of bacterial communities in activated sludge systems [20-22] but not much is known about the archaeal communities present in sludge digester operating in the UAE. As per our knowledge, there hasn’t been any study published so far regarding the community structure of methanogenic Archaea inhabiting the anaerobic digesters of full-scale Wastewater Treatment Plant (WWTP) in the UAE. Therefore, this study attempts to provide insights into the methanogenic consortia and analyze the different methanogenic groups present.

## 2 MATERIALS AND METHODS

### 2.1 Anaerobic Digester Sampling

The waste sludge samples were collected from the Jebel Ali Wastewater Treatment Plant (JASTP), Dubai, UAE on a monthly basis for a period of five months. JASTP is one of the two-wastewater treatment facilities in the emirate of Dubai with the capacity of treating about 375,000 cubic meters of mostly domestic wastewater on daily basis. JASTP utilize activated sludge process coupled with advanced level nitrogen removal stages. The treated effluent from biological stages undergoes further treatment by sand filtration and UV based disinfection. The large quantities of sludge produced during various stages of wastewater treatment process pass through full-scale sludge digesters where anaerobic digestion of sludge take place during which sludge is stabilized and part of solids are converted to methane gas. The tertiary treated effluent is reused in irrigation, and treated sludge is converted to manure for use as soil conditioner and fertilizer.

There are five full-scale continuous stirred type anaerobic digesters in total but for this project the samples were collected from anaerobic digesters 1, 3 & 5. These three digesters were chosen for this study as per recommendation from the treatment plant officials based on construction timing of the digesters. The digester 3 is the oldest and digester 5 (the newest) and digester 1 was constructed sometime between to the 3 and 5. The operating physicochemical conditions data of the digesters was provided by Jebel Ali Sewage Treatment Plant laboratory, Dubai, UAE Table **1**. The samples were directly taken from anaerobic digesters (1, 3 & 5) into an autoclaved plastic bottle with 1 liter volume. The sample bottles were placed in an icebox and brought to the laboratory. The collected samples were stored at 4^o^C until DNA extraction and fixation of biomass for qPCR and FISH analysis, respectively.

### 2.2 Fluorescence *in situ* Hybridization

The samples were fixed within 24 hours of sampling with paraformaldehyde; incubated for 3-4 hours and then washed thrice with 1X PBS (Phosphate Buffer Saline) and stored at -20^o^C. FISH is a method basically used to quantify the presence and relative abundance of targeted microbes within the sample. It includes three major steps firstly, the hybridization of the cells with the probes, followed by washing off the excessive probe finally the visualization of the hybridized cells under the fluorescence microscope as per the standard procedure described earlier (26-28).

For the hybridization, teflon printed slides with wells of 8mm diameter from Vermicon VIT identification technology (Munich, Germany) were used. The slides were cleaned with acid alcohol (1% HCl in 70% EtOH) and placed in 0.01% Poly-L-Lysine solution for adhering to the fixed cells.

1 – 3μl of the sample was applied to a coated slide and air-dried. These cells were then dehydrated using a graded ethanol series (50%, 80%, 96% for 3 minutes each) and air dried for 5 minutes. Subsequently, the probe mix was prepared by adding 9μl of the hybridization solution and 1μl of the respective working probe. The remainder of the hybridization solution was loaded onto the tray of the polypropylene hybridization chamber and placed in the oven at 46^o^C. The probe mix was applied to the respective well on the slide. The slide was then placed in the hybridization chamber with the tray containing hybridization solution. The chamber was tightly sealed and incubated at 46^o^C for 3 – 4 hours. The different probes targeted for 16S rRNA used for this study are listed in Table **2**. All the oligonucleotide probes were labeled with cyanine dye Cy3.

The washing solutions were prepared that accompanied the hybridization solutions used earlier. The washing solution was pre-warmed at 48^o^C. The slide was rinsed with 1ml of pre-warmed washing solution. Then slides were kept immersed in the washing solution and incubated at 48^o^C for 10-15 mins. Post incubation, the slide was rinsed with ddH_2_O at room temperature, air dried for a few minutes and visualized under the fluorescence microscope. Prior to visualization, 3μl of 10 mg/l DAPI (4',6-diamidino-2-phenylindole) was added to each well. The slide was then visualized under the fluorescence microscope system Olympus BX51 Series which was connected to a DP72 digital camera. Images were captured using the DP2-BSW Software. Images taken from the microscope were subjected to MetaMorph software (Version 7.10.0.119 to count the number of cells present in each image. For each sample minimum of at least 10 images of same DAPI and CY3 images were taken and then considered the average of all to calculate the total number of cells versus the % of hybridized cells.

### 2.3 Quantitative Polymerase Chain Reaction (qPCR)

Genomic DNA was extracted from the sludge samples using Power Soil DNA Extraction Kit (MO BIO Laboratories). The samples were vortexed and then subsequently centrifuged to get rid of maximum water content. DNA was then extracted from 0.25g of the obtained pellet according to the manufacturer’s protocol. The solution containing the extracted DNA was stored at -20^o^C to -80^o^C. The A_260_/A_280_ and A_260_/A_230_ ratios were utilized to determine the purity and concentration of the extracted DNA using a Nano-Drop 2000c spectrophotometer (Thermo Fisher Scientific, USA). Quantitative PCR (qPCR) was employed to relatively quantify the presence of the respective methanogenic archaeal members relative to an endogenous control using the Comparative C_T_ method (∆∆C_T_). The endogenous control used in this study was archaeal bacterial DNA extracted from the digester sludge and amplified using archaeal domain specific primer and probe sets described in Table **3**. The qPCR amplifications were performed in 20μl reactions. Each reaction contained 1μl of 20X stock assay (5μl of 10μM forward/reverse primer, 5μl of respective 5 μM TaqMan probe, 85μl of PCR-grade pure water), 10μl of TaqMan Master Mix, 8μl of PCR grade pure water and 1μl of extracted template DNA. Two-step amplification of the target DNA, combining the annealing and the extension steps, was performed applying the following conditions an initial 10-min incubation at 95^o^C for denaturation & Taq DNA polymerase activation followed by 40 cycles of denaturation at 95^o^C for 15s; and simultaneous annealing and extension at 60^o^C for 1min. Methanogens, the key players responsible for methanogenesis, were investigated at the domain, 4 different order and 2 family levels covering majority of the methanogenic archaea present in anaerobic digester systems to obtain a comprehensive insight into their community structures. Real-Time PCR was performed using StepOnePlus™ Real-Time PCR System (Applied Biosystems, USA) with seven primer and probe sets listed in the Table (**3**).

### 2.4 Result & Discussion

#### 2.4.1 Profiling of Archaeal Community Composition by FISH Technique

The JASTP consists of five full-scale anaerobic digesters. For this study, sludge samples were obtained from AD 1, 3 & 5. The three digesters in sequence of their age, newest to oldest, are AD5, AD1 & AD3. Under optimal hybridization conditions, methanogenic Archaea were specifically visualized and detected using the corresponding probes labeled with Cy3. Fig. (**1**) displays the epifluorescence micrograph showing *in situ* hybridization with probe ARC915. In each respective sludge sample, the active Archaeal populations were observed with respect to 4’, 6-diamidino-2-phenylindole (DAPI) [26-28].

Yellow color oval represents Coccus, blue color for clumps of filaments green color represents rod-shaped cell.

Samples were hybridized with the universal Archaeal probe ARC915 as well as with order-, family- and genus-specific 16S rRNA oligonucleotide probes. A large majority of the Archaeal community gave positive hybridization signals with the ARC915 probe. From the photomicrographs it was observed that 49.73%, 47.72%, 54.13% of cells belonged to the archaea for the anaerobic digester AD1, AD3, and AD5 respectively when compared with the total number of cells. This probe identified various archaeal morphologies like cocci, rod-shaped cells and clumps of long filaments as shown in Fig. (**2**).

For samples taken from anaerobic digester 1, 3 and 5, probes targeting the subgroups of class *Methanomicrobia* were utilized: MG1200b, MS1414, MS821, MX1361 and MX825. Two probes, *i.e.*, MX1361 and MX825 were employed to target the family *Methanosaetaceae* to compare the result of hybridized cells targeted by these two probes. Upon comparison, it was observed that overall, MX825 hybridized with a greater percentage of the target Methanosaetaceae cells than probe MX1361. This observation is supported by the finding of the study conducted by Raskin *et al.* 1996 [23], wherein it is stated that probe MX825 should be used only for the characterization of microbial communities under mesophilic conditions. According to Crocetti *et al.* 2006 [24], the accurate specificity of probe MX1361 cannot be appreciated due to the fact that GenBank houses comparatively scarce sequence data for MX1361 target position.

In both AD1 and AD3, the family *Methanosaetaceae* (MX825) and in AD5, the family *Methanosarcinaceae* was the predominant Archaeal methanogens contributing 17%, 17% and 16% respectively Fig. (**2**) among the total Archaeal population. The existence of the *Methanosaetaceae* family members has been extensively reported in anaerobic bioreactors. In addition, the presence of the species *Methanosaeta* has been linked to a more stable and consistent bioreactor operation [29-31]. In all the samples, the MX825 and MX1361 probes identified rod-shaped cells either present individually or forming a chain of rods. Within the granule, these rods appear to play an instrumental role in contributing to a network thereby facilitating the association of other bacteria to this network [32]. Filamentous like aggregations of *Methanosaeta* cells were also observed. Garrity and Holt, in 2001 [33] have reported the formation of filaments comprising of 10-300 cells by *Methanosaeta* species. According to Zitomer [34], the filamentous morphology of Methanosaeta aid in the process of granule formation wherein the filaments serve as binders to help hold the granule together however granulation process is not relevant as all digesters were continuously stirred type reactors. In some of the samples, quite a few randomly distributed cocci were also noticed. It is difficult to confirm whether these cells belong to the targeted family since only rod shaped and filamentous morphology has been observed previously for the family Methanosaetaceae members.

On the other hand, probes MS1414 and MS821 targeting family *Methanosarcinaceae* and genus *Methanosarcina* respectively identified numerous coccoidal cells. These cells assumed various patterns like pairs, chain and irregular clumps. According to Demirel and Scherer [35], existence of these cells in clumps aid in their protection from harmful chemical agents. Formation of irregular cell clumps by members of the genus *Methanosarcina* increases their tolerance to high concentrations of toxic ionic agents and pH fluctuations [36]. The second populous Archaeal group belonged to genus *Methanosarcina* (MS821) in AD1 and AD3 (15% each) whereas in AD5 it belonged to order *Methanomicrobiales* (15%) from the total archaeal population.

Previous culture-independent studies have shown the families *Methanosarcinaceae* and *Methanosaetaceae* (in the order *Methanosarcinales*) and the order *Methanomicrobiales* to be very common in anaerobic digestion [37]. All members of the order *Methanomicrobiales* produce methane by CO_2_ reduction with H_2_ whereas the order *Methanosarcinales* use acetate as the sole substrate to carry out methanogenesis [38]. Moreover, acetate is a more common substrate than CO_2_ methanogenesis since wastewater usually contain high levels of organic acids [39]. This could be a plausible explanation for their prevalence in all the three anaerobic digesters.

The order *Methanomicrobiales* exhibited a nearly similar hybridization percentage *i.e.*, 12% and 11% in AD1 and AD3 respectively. Only in digester 5, the percentage shot up to 15% suggesting a greater abundance in this digester. The members of this order targeted by oligonucleotide probe MG1200b displayed diverse morphologies including small cocci and rods and many filamentous shaped Archaeal cells. This morphological observation is in line with the literature discussed by Seckbach [40], wherein this order was reported to include rods, cocci, irregular cocci, ring- or corpuscle-shaped organisms, plates and spirals.

The predominance of class *Methanomicrobia* is associated with the abundant methanogens in the sample, in which numerous *Methanosarcinale* members were detected. Oligonucleotide probes MB1175 and MB310 were used to target the *Methanobacteria* subgroups at the order and family level respectively. MB1175 succeeded in hybridizing with 10%, 14% and 13% of the target cells whereas MB310 hybridized with 12%, 10% and 8% of the target cells belonging to family *Methanobacteriaceae* in AD1, AD3 and AD5 out of the total archaeal population. These numbers make it quite evident that the methanogenic population contributed by the class *Methanobacteria* was comparatively lower than class *Methanomicrobia*. Members of the order *Methanobacteriales* are generally hydrogenotrophic, which utilize a narrow range of substrates for methanogenesis, which include H_2_, CO_2_ and formate [38]. The cells identified by the above-mentioned probes displayed two main morphologies: a) cocci shaped cells existing alone or in pairs (diplococci) b) rod-shaped cells forming chains & clumps of filaments. Seckbach and Boone [40, 41] described the order Methanobacteriales to mainly consist of short rods. However, we have observed the presence of few coccoidal cells existing either alone or in pairs.

The MC1109 probe targeting *Methanococcales* detected 10% each for AD1 and AD3; AD5 showed 7% of the target irregular cocci cells among the total archaeal cells. This suggests that very few cells of this order were present indicating that *Methanococcale* type-methanogens were not frequent in mesophilic digesters. Members of this order use H_2_ or formate to reduce CO_2_ for methanogenesis [38].

Overall upon close observation, it is quite evident that the *Methanosaetacea*, *Methanosarcinacea* and *Methanomicrobia* subgroups occupied the top positions in terms of dominance in nearly all the three anaerobic digesters. The presence of aceticlastic methanogens outweighs the hydrogenotrophic methanogens in nearly all the three anaerobic digesters suggesting that the aceticlastic pathway is probably the favorable route for methanogenesis in these digesters. Moreover, aceticlastic methanogens have been reported to be responsible for approximately 70% of the methane produced in anaerobic digesters [42, 43].

#### 2.4.2 Analysis of Methanogenic Community by Real-Time Quantitative PCR

Quantitative real time polymerase chain reaction (qPCR) has gained popularity in recent years as a method for determining microbial populations in methanogenic systems [17, 18, 44]. In this study, quantitative real time PCR was applied for the relative quantification of predominant methanogenic Archaea at the level of domain, order and family by the Comparative C_T_ method. qPCR was performed for sludge samples of 3 months *i.e.*, November, December and January. For this comparative C_T_ study, the Archaeal 16S rRNA was chosen as the endogenous control.

To use qPCR to quantify rRNA, the computer software connected to the instrument constructs the amplification plot for detecting increase in fluorescence emission. The fluorescence emission generated and detected as a threshold cycle (C_T_) value. The C_T_ value is defined as the actual PCR cycle when the intensity of the fluorescent signal increases to above the background threshold and is proportional to the initial copy number of the target gene. For use of the same primer and probe sets for the target group, the higher the C_T_ value, the lower the initial rRNA concentration is likely to be [7]. For all the samples analyzed, the amplification plot and C_T_ values obtained for the targeted orders and families are depicted in the Fig. (**3**).

In the month of November, AD1 and AD3 had exactly the same trend of Archaeal population *i.e.*, *Methanosaetaceae* (MST) followed by *Methanobacteriales* (MBL), *Methanomicrobiales* (MMB), *Methanosarcinales* (MSL) and *Methanosarcinaceae* (MSC). However, in AD5, order *Methanomicrobiales* and *Methanosarcinales* were detected more than *Methanobacteriales*.

In the month of December, AD3 and AD5 consisted of methanogenic Archaea that populated the digesters in the same sequence *i.e.*, *Methanosaetaceae* followed by *Methanomicrobiales*, *Methanosarcinales*, *Methanobacteriales* and *Methanosarcinaceae*. AD1 also displayed a similar trend with slight variations: a) MMB was slightly higher in abundance than MST b) MBL was the least abundant. In December and January, the archaeal communities in a similar range populated AD3 and AD5. AD1 showed a slight difference with *Methanobacteriales* dominating over *Methanosarcinales*. This indicates that overall the family *Methanosaetaceae* was the predominant taxon whereas the family *Methanosarcinaceae* was the least abundant throughout the months of November, December and January. Comparing the two-targeted orders, *Methanosarcinales* prevailed over *Methanomicrobiales* although the latter wasn’t far behind in terms of C_T_ value. The MCL primer and probe set failed to detect the members of the order *Methanococcales* in this trial. This could be either because the members of this order were below the detection limit of this technique or probably due to their growth requirement of high salt conditions (0.3-9.4% (w/v) NaCl) that are not normally found in anaerobic reactors [41]. The results obtained using the two molecular techniques *i.e.*, FISH and qPCR were compared. According to qPCR, at the order level, *Methanomicrobiales* seems to be predominant in the months of December and January in all the three anaerobic digesters whereas in November AD1 and AD3 were dominated by order *Methanobacteriales*. However, FISH results indicate that *Methanomicrobiales* dominated AD1 and AD5 during the three months whereas order *Methanobacteriales* prevailed in AD3. The increased abundance of Methanomicrobiales might be hypothetically correlated with the presence of more diverse bacterial communities [32].

Both FISH and qPCR data suggested that at the family level, barring AD1 in December, *Methanosaetaceae* was the most abundant throughout the three-month period in nearly all the digesters while *Methanosarcinaceae* was the least prevalent. Most of the Methanosaetaceae family members survive best at pH between 6.6 – 7.5 and temperature between 30^o^C – 40^o^C. The operational parameters of the anaerobic digesters under study seem to suit their growth requirements and favor their proliferation causing them to be prevailing in these digesters. The findings of the study conducted by Karakashev and colleagues [45] indicated that the methanogenic diversity was broader in plants operating at mesophilic ranges than the thermophilic plants. The research study conducted by Liu *et.al.* in 2002 [46], proposed that abundant Methanosaeta spp. improves granulation and consequently leads to more stable reactor performance. However, granulation process is not relevant to the observed predominance of Methanosaetaceae members as studied anaerobic digesters were continuously stirred tank type reactors. Overall, the results obtained by the two molecular techniques seem to agree at the family level but not much at the order level. Previous studies suggest that qPCR results are considered to be more efficient and superior to FISH technique [47].

## CONCLUSION

The aim of this study was to evaluate the methanogenic archaeal community structure of three full-scale anaerobic digester systems of a municipal wastewater treatment plant in Dubai, UAE. The fluorescence *in situ* hybridization and quantitative real-time PCR was used for *in-situ* identification and quantification of the methanogenic archaeal community in the anaerobic digesters. The archaeal populations were targeted at the domain, order and family level. All the three anaerobic digesters showed almost similar type of Archaeal community. The results of this study suggest the dominance of the family Methanosaetaceae in all the digesters. These results suggest that the methane in these digesters is produced through aceticlastic methanogenesis. Further work to obtain in-depth understanding of the relationships between archaeal communities, their functional gene under the influence of key operational physico-chemical parameters using high-throughput sequencing methods will help to provide better understanding of the dynamics of the methanogenic archaeal community in wastewater treatment plants in UAE and this knowledge will help to improve the performance of anaerobic digesters.

## Figures and Tables

**Fig. (1):**
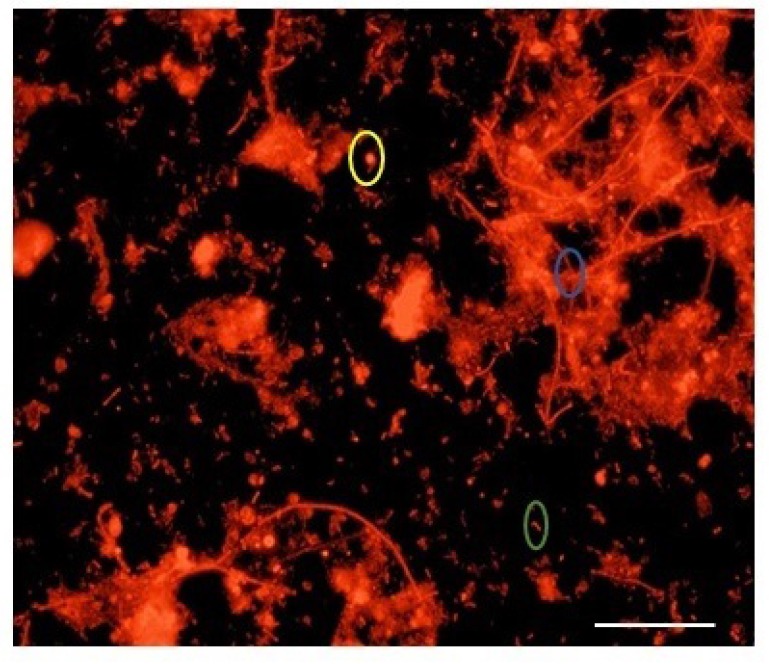
Epifluorescence micrograph showing *in situ* hybridization with probe ARC915 – Cy3; Bar = 10μm.

**Fig. (2):**
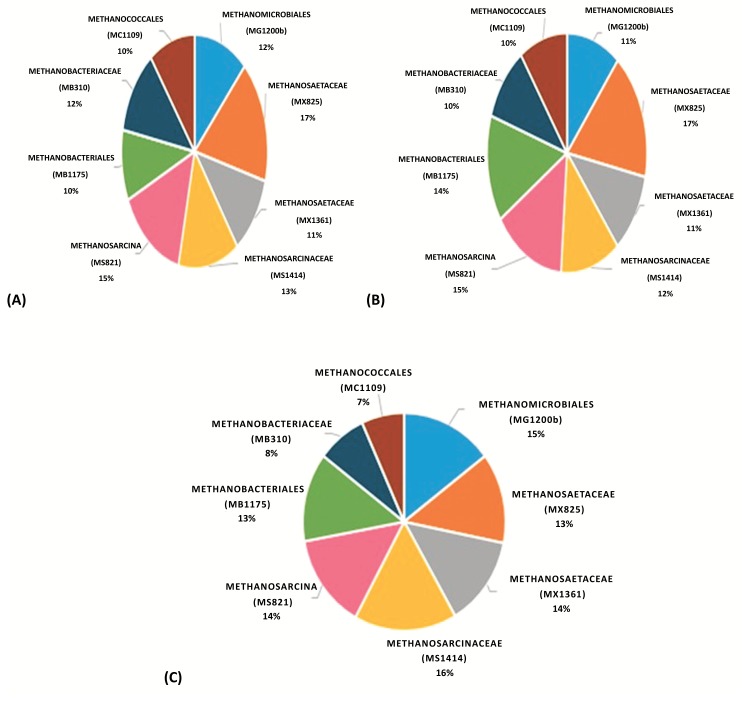
The proportions contributed by various methanogenic Archaea versus total Archaeal population. (A) Anaerobic Digester-1 (AD1) (B) Anaerobic Digester-3 (AD3) (C) Anaerobic Digester-5 (AD5)

**Fig. (3):**
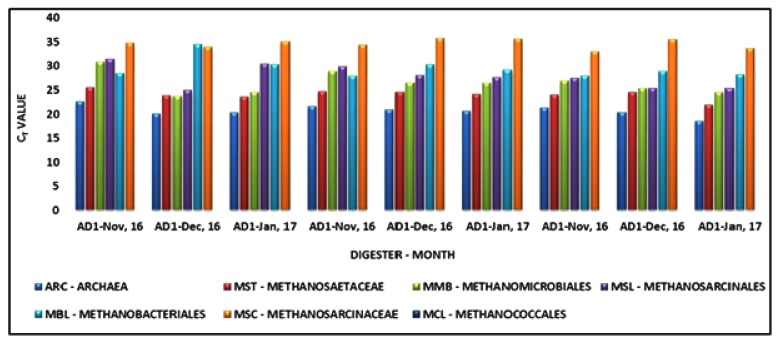
C_T_ values of probes targeted at mehanogenic community in sludge digesters over sampling period.

**Table 1:** Operational physicochemical conditions of the anaerobic sludge digesters.

**Digester Feed**	**60% Raw Sludge, 40% Activated Sludge**		
Digester Number	**1**	**3**	**5**
**Parameters**			
Digester capacity (m^3^)	7433	7433	7433
pH	7.13 -7.5	7.27-7.55	7.36
Temperature(^0^C)	34	34	34
Digester feeding per day (m^3^)	2248	2148	2552
Solid retention time (days)	16	16	14
Up flow velocity (m^3^/hr)	120	120	120
Hydraulic Retention Time (HRT) (days)	3.3	3	2.91
Organic loading rate (kg. oDS / m^3^.d)	6.84	5.84	6.61
Dry solid (%)	2.91-3.34	2.56-5.74	2.79-3.54
Volatile solids (%)	70.27-70.95	43.75-70.15	54.54-67.49
Volatile fatty acid	165-195	168-205	145.5-195
Alkalinity	3014-3451	2992-3512	2893-3190
Dissolved sulfide (mg/L)	37.2-38	32.4-37.2	26.4-27.6

**Table 2:** Oligonucleotide probes used in this study.

**Probe**	**Sequence (5’ – 3’)**	**Target**	**Rank**	**Formamide %**	**References**
ARC915	GTGCTCCCCCGCCAATTCCT	Most *Archaea*	DOMAIN	35	[23]
MG1200b	CRGATAATTCGGGGCATGCTG	Most *Methanomicrobiales*	ORDER	20	[24]
MX825	TCGCACCGTGGCCGACACCTAGC	*Methanosaetaceae*	FAMILY	50	[23]
MS1414	CTCACCCATACCTCACTCGGG	*Methanosarcinaceae*	FAMILY	50	
MS821	CGCCATGCCTGACACCTAGCGAGC	Some *Methanosarcina*	GENUS	40	
MC1109	GCAACATAGGGCACGGGTCT	*Methanococcales*	ORDER	45	
MB1175	TACCGTCGTCCACTCCTTCCTC	Most *Methanobacteriales*	ORDER	45	
MB310	CTTGTCTCAGGTTCCATCTCCG	*Methanobacteriales*	ORDER	35	
MX1361	ACGTATTCACCGCGTTCTGT	*Methanosaetaceae*	FAMILY	25	[24]

**Table 3:** Primer and probe sets used for qPCR.

**Primer set**	**Function**	**Target group**	**Rank**	**Sequence (5’ –3’)**	**References**
ARC	F. Primer	*Archaea*	DOMAIN	ATTAG ATACC CSBGT AGTCC	[7]
	TaqMan			AGGAA TTGGC GGGGG AGCAC	
	R. Primer			GCCAT GCACC WCCTC T	
MCL	F. Primer	*Methanococcales*	ORDER	TAAGG GCTGG GCAAG T	[25]
	TaqMan			TAGCG GTGRA ATGYG TTGAT CC	
	R. Primer			CACCT AGTYC GCARA GTTTA	
MBL	F. Primer	*Methanobacteriales*	ORDER	CGWAG GGAAG CTGTT AAGT	
	TaqMan			AGCAC CACAA CGCGT GGA	
	R. Primer			TACCG TCGTC CACTC CTT	
MMB	F. Primer	*Methanomicrobiales*	ORDER	ATCGR TACGG GTTGT GGG	[7]
	TaqMan			TYCGA CAGTG AGGRA CGAAA GCTG	
	R. Primer			CACCT AACGC RCATH GTTTA C	
MSL	F. Primer	*Methanosarcinales*	ORDER	GTAAA CGATR YTCGC TAGGT	
	TaqMan			AGGGA AGCCG TGAAG CGARC C	
	R. Primer			GGTCC CCACA GWGTA CC	
MSC	F. Primer	*Methanosarcinaceae*	FAMILY	GAAAC CGYGA TAAGG GGA	
	TaqMan			TTAGC AAGGG CCGGG CAA	
	R. Primer			TAGCG ARCAT CGTTT ACG	
MST	F. Primer	*Methanosaetaceae*	FAMILY	TAATC CTYGA RGGAC CACCA	
	TaqMan			ACGGC AAGGG ACGAA AGCTA GG	
	R. Primer			CCTAC GGCAC CRACM AC	

## LIST OF ABBREVIATIONS

WWTP: = Wastewater Treatment Plant
JASTP: = Jebel Ali Sewage Treatment Plant
FISH: = Fluorescence *in situ* hybridization
qPCR: = real-time quantitative Polymerase Chain Reaction
AD: = Anaerobic Digester
C_T_: = Threshold cycle
T-RFLP: = Terminal Restriction Fragment Length Polymorphism
DGGE: = Denaturing Gradient Gel Electrophoresis
SSCP: = Single Strand Conformation Polymorphism

## References

[1] Narihiro T., Sekiguchi Y. (2007). Microbial communities in anaerobic digestion processes for waste and wastewater treatment: a microbiological update.. Curr. Opin. Biotechnol..

[2] Hufnagel D., Chang S., Zhang J., Lu R. (2015). Analysis of methanogen communities in anaerobic membrane bioreactors and conventional anaerobic digester systems.. JAAB.

[3] Kim J., Kim W., Lee C. (2013). Absolute dominance of hydrogenotrophic methanogens in full-scale anaerobic sewage sludge digesters.. J. Environ. Sci. (China).

[4] Connaughton S., Collins G., O’Flaherty V. (2006). Development of microbial community structure and actvity in a high-rate anaerobic bioreactor at 18°C.. Water Res..

[5] Costa K.C., Leigh J.A. (2014). Metabolic versatility in methanogens.. Curr. Opin. Biotechnol..

[6] Borrel G., O’Toole P.W., Harris H.M., Peyret P., Brugère J.F., Gribaldo S. (2013). Phylogenomic data support a seventh order of Methylotrophic methanogens and provide insights into the evolution of Methanogenesis.. Genome Biol. Evol..

[7] Yu Y., Lee C., Kim J., Hwang S. (2005). Group-specific primer and probe sets to detect methanogenic communities using quantitative real-time polymerase chain reaction.. Biotechnol. Bioeng..

[8] Yu Y., Kim J., Hwang S. (2006). Use of real-time PCR for group-specific quantification of aceticlastic methanogens in anaerobic processes: population dynamics and community structures.. Biotechnol. Bioeng..

[9] Sekiguchi Y. (2006). Yet-to-be cultured microorganisms relevant to methane fermentation processes.. Microbes Environ..

[10] Sakai S., Imachi H., Sekiguchi Y., Tseng I.C., Ohashi A., Harada H., Kamagata Y. (2009). Cultivation of methanogens under low-hydrogen conditions by using the coculture method.. Appl. Environ. Microbiol..

[11] Díaz E.E., Stams A.J., Amils R., Sanz J.L. (2006). Phenotypic properties and microbial diversity of methanogenic granules from a full-scale upflow anaerobic sludge bed reactor treating brewery wastewater.. Appl. Environ. Microbiol..

[12] Ennouri H., Miladi B., Diaz S.Z., Güelfo L.A.F., Solera R., Hamdi M., Bouallagui H. (2016). Effect of thermal pretreatment on the biogas production and microbial communities balance during anaerobic digestion of urban and industrial waste activated sludge.. Bioresour. Technol..

[13] Zahedi S., Solera R., Micolucci F., Cavinato C., Bolzonella D. (2016). Changes in microbial community during hydrogen and methane production in two-stage thermophilic anaerobic co-digestion process from biowaste.. Waste Manag..

[14] Akuzawa M., Hori T., Haruta S., Ueno Y., Ishii M., Igarashi Y. (2011). Distinctive responses of metabolically active microbiota to acidification in a thermophilic anaerobic digester.. Microb. Ecol..

[15] Watanabe T., Asakawa S., Nakamura A., Nagaoka K., Kimura M. (2004). DGGE method for analyzing 16S rDNA of methanogenic archaeal community in paddy field soil.. FEMS Microbiol. Lett..

[16] Sawayama S., Tsukahara K., Yagishita T. (2006). Phylogenetic description of immobilized methanogenic community using real-time PCR in a fixed-bed anaerobic digester.. Bioresour. Technol..

[17] Williams J., Williams H., Dinsdale R., Guwy A., Esteves S. (2013). Monitoring methanogenic population dynamics in a full-scale anaerobic digester to facilitate operational management.. Bioresour. Technol..

[18] Koo T., Shin S.G., Lee J., Han G., Kim W., Cho K., Hwang S. (2017). Identifying methanogen community structures and their correlations with performance parameters in four full-scale anaerobic sludge digesters.. Bioresour. Technol..

[19] Delbès C., Moletta R., Godon J. (2001). Bacterial and archaeal 16S rDNA and 16S rRNA dynamics during an acetate crisis in an anaerobic digestor ecosystem.. FEMS Microbiol. Ecol..

[20] Juretschko S., Loy A., Lehner A., Wagner M. (2002). The microbial community composition of a nitrifying-denitrifying activated sludge from an industrial sewage treatment plant analyzed by the full-cycle rRNA approach.. Syst. Appl. Microbiol..

[21] Figuerola E.L., Erijman L. (2007). Bacterial taxa abundance pattern in an industrial wastewater treatment system determined by the full rRNA cycle approach.. Environ. Microbiol..

[22] Bouchez T., Jacob P., d’Hugues P., Durand A. (2006). Acidophilic microbial communities catalyzing sludge bioleaching monitored by fluorescent in situ hybridization.. Antonie van Leeuwenhoek.

[23] Raskin L., Stromley J.M., Rittmann B.E., Stahl D.A. (1994). Group-specific 16S rRNA hybridization probes to describe natural communities of methanogens.. Appl. Environ. Microbiol..

[24] Crocetti G., Murto M., Björnsson L. (2006). An update and optimisation of oligonucleotide probes targeting methanogenic Archaea for use in fluorescence in situ hybridisation (FISH).. J. Microbiol. Methods.

[25] Kim T.G., Jeong S.Y., Cho K.S. (2015). Development of droplet digital PCR assays for methanogenic taxa and examination of methanogen communities in full-scale anaerobic digesters.. Appl. Microbiol. Biotechnol..

[26] Manz W., Amann R., Ludwig W., Wagner M., Schleifer K. (1992). Phylogenetic oligodeoxynucleotide probes for the major subclasses of proteobacteria: Problems and solutions.. Syst. Appl. Microbiol..

[27] Amann R., Fuchs B.M., Behrens S. (2001). The identification of microorganisms by fluorescence in situ hybridisation.. Curr. Opin. Biotechnol..

[28] Daims H., Stoecker K., Wagner M., Osborn M., Smith C. (2005). Fluorescence in situ hybridization for the detection of prokaryotes.. Advanced methods in molecular microbial ecology..

[29] Chan O.C., Liu W.T., Fang H.H. (2001). Study of microbial community of brewery-treating granular sludge by denaturing gradient gel electrophoresis of 16S rRNA gene.. Water Sci. Technol..

[30] Batstone D.J., Keller J., Blackall L.L. (2004). The influence of substrate kinetics on the microbial community structure in granular anaerobic biomass.. Water Res..

[31] Hulshoff Pol L.W., de Castro Lopes S.I., Lettinga G., Lens P.N. (2004). Anaerobic sludge granulation.. Water Res..

[32] Bialek K., Kim J., Lee C., Collins G., Mahony T., O’Flaherty V. (2011). Quantitative and qualitative analyses of methanogenic community development in high-rate anaerobic bioreactors.. Water Res..

[33] Garrity G., Holt J., Boone D., Castenholz R. (2011). Phylum AII. Euryarchaeota.. Bergey’s Manual of Systematic Bacteriology..

[34] Zitomer D, Maki J, Venkiteshwaran K, Bocher B. (2016). Relating Anaerobic Digestion Microbial Community and Process Function.Microbiol Insights.

[35] Demirel B., Scherer P. (2008). The roles of acetotrophic and hydrogenotrophic methanogens during anaerobic conversion of biomass to methane: a review.. Rev. Environ. Sci. Biotechnol..

[36] Conklin A., Stensel H.D., Ferguson J. (2006). Growth kinetics and competition between Methanosarcina and Methanosaeta in mesophilic anaerobic digestion.. Water Environ. Res..

[37] Steinberg L.M., Regan J.M. (2008). Phylogenetic comparison of the methanogenic communities from an acidic, oligotrophic fen and an anaerobic digester treating municipal wastewater sludge.. Appl. Environ. Microbiol..

[38] Timmis K. (2010). Handbook of hydrocarbon and lipid microbiology..

[39] Microbewiki.kenyon.edu (2017).

[40] Seckbach J. (2013). Journey to Diverse Microbial Worlds. Dordrecht..

[41] Boone D., Castenholz R. (2001). The Archaea and the deeply branching and phototrophic bacteria.. Bergey’s Manual of Systematic Bacteriology..

[42] Jetten M. (1992). Methanogenesis from acetate: a comparison of the acetate metabolism in Methanothrix soehngenii and Methanosarcina spp.. FEMS Microbiol. Lett..

[43] Ahring B.K. (1995). Methanogenesis in thermophilic biogas reactors.. Antonie van Leeuwenhoek.

[44] Bartell R.D., Matson E., Mueller-Spitz S., Kleinheinz T.G. (2015). Investigation of methanosarcinales and methanomicrobiales presence within a dry Anaerobic digester.. J. Microbiol. Res. (Rosemead Calif.).

[45] Karakashev D., Batstone D.J., Angelidaki I. (2005). Influence of environmental conditions on methanogenic compositions in anaerobic biogas reactors.. Appl. Environ. Microbiol..

[46] Liu Y., Xu H., Show K., Tay J. (2002). Anaerobic granulation technology for wastewater treatment.. World J. Microbiol. Biotechnol..

[47] Ramdhani N., Kumari S., Bux F. (2014). Comparison of quantitative PCR and fluorescent in situ hybridization techniques to quantify nitrobacter from wastewater samples.. IWA World Water Congress Secretariat.

